# Cell segmentation for immunofluorescence multiplexed images using two-stage domain adaptation and weakly labeled data for pre-training

**DOI:** 10.1038/s41598-022-08355-1

**Published:** 2022-03-15

**Authors:** Wenchao Han, Alison M. Cheung, Martin J. Yaffe, Anne L. Martel

**Affiliations:** 1 Biomarker Imaging Research Laboratory, Sunnybrook Research Institute, Toronto, ON Canada; 2 Department of Medical Biophysics, University of Toronto, Toronto, ON Canada

**Keywords:** Cancer, Cell biology, Engineering

## Abstract

Cellular profiling with multiplexed immunofluorescence (MxIF) images can contribute to a more accurate patient stratification for immunotherapy. Accurate cell segmentation of the MxIF images is an essential step. We propose a deep learning pipeline to train a Mask R-CNN model (deep network) for cell segmentation using nuclear (DAPI) and membrane (Na^+^K^+^ATPase) stained images. We used two-stage domain adaptation by first using a weakly labeled dataset followed by fine-tuning with a manually annotated dataset. We validated our method against manual annotations on three different datasets. Our method yields comparable results to the multi-observer agreement on an ovarian cancer dataset and improves on state-of-the-art performance on a publicly available dataset of mouse pancreatic tissues. Our proposed method, using a weakly labeled dataset for pre-training, showed superior performance in all of our experiments. When using smaller training sample sizes for fine-tuning, the proposed method provided comparable performance to that obtained using much larger training sample sizes. Our results demonstrate that using two-stage domain adaptation with a weakly labeled dataset can effectively boost system performance, especially when using a small training sample size. We deployed the model as a plug-in to CellProfiler, a widely used software platform for cellular image analysis.

## Introduction

Immuno-oncology profiling requires a detailed assessment of the tumor microenvironment^1^. This includes identifying and quantifying different immune cell subsets, their spatial arrangement, and the expression of immune checkpoint markers on these cells^2^. Simultaneously characterizing both immune and tumor-related pathways can empower a more accurate patient stratification for immunotherapy^3–5^. Advances in imaging and automatic analysis (including artificial intelligence) can dramatically impact the ability to perform such characterization^1^. Immunofluorescent multiplexing, one of several multiplexing technologies that have recently become available, allows the labeling of different protein markers with immunofluorescent (IF) conjugated antibodies on the same tissue section simultaneously. Accurate cell segmentation on the multiplexed immunofluorescence (MxIF) images is an essential step when generating profiling information for downstream analysis.

Meijering^6^ published a comprehensive review of the literature on methodologies for nuclear/cell segmentation, covering many conventional algorithms (i.e. non-deep learning), including thresholding, filtering, morphological operations, region accumulation, and model fitting. These methods used alone are seldom able to achieve satisfactory results. More commonly, combinations of methods are used for specific nuclear/cell segmentation tasks. For example, Veta et al.^7^ proposed a pipeline that used maker-controlled watersheds followed by postprocessing steps to segment cell nuclei on haematoxylin and eosin (H&E) stained images. They used color unmixing, morphological operations followed by a fast radial symmetry transform^8^ to extract foreground and background markers to perform the marker-controlled watershed algorithm^9^. Software platforms have been developed to make it easier to adapt existing methods to new tasks^10,11^. For example, CellProfiler^11^ provides a platform for the user to construct a pipeline by selecting one or more conventional algorithms. Specifically, it provides a graphical user interface (GUI), where users can select algorithms and place them in a serial order by dragging them into the pipeline. Since those conventional algorithms usually have low computation cost, they are easy to deploy, which makes them popular and widely used. However, there are some disadvantages of this approach: (1) the use of these methods usually involves manually fine-tuning parameters, which is often labor-intensive and time-consuming^12^, (2) methods built from those conventional algorithms often do not generalize well for batch processing or processing images from different datasets, (3) they may not be able to achieve satisfactory performance for application^6^.

Recently, deep learning, specifically fully convolutional networks (FCN)^13^ based methods, such as U-Net^14^, DoGnet^15^, and DeepCell^16^ have come into wide use for cell/nuclear segmentation. They demonstrate better accuracy in performance for cell nuclear segmentation^14,16,17^ with much better generalization capacity and are suitable for batch processing. Since the core concept of deep learning is to train a model using labeled samples, a large quantity of labeled data that covers an appropriately broad range of sample variation is necessary to achieve satisfactory results.

The above-mentioned methods utilize semantic segmentation, i.e. a label is assigned to each pixel and the object-level information is not necessarily preserved. For example, the neighbouring cells may not be separated, and they may be considered as one unit. In contrast, instance segmentation identifies each instance (i.e. cell) and provides a partitioning^18^. Semantic segmentation followed by various post processing steps can be used to achieve instance segmentation. In the cellular image segmentation task, for example, instance segmentation can be accomplished by classifying each pixel as either nuclear/cell interior, edge, or background and then using a connected region growing method to label each interior instance. It is often the case that there are crowded, touching cells/nuclei, and cells/nuclei that have staining variation. This makes the isolation of individual cells/nuclei difficult. For those methods, post processing steps^14,15^ for grouping pixel labels, splitting touching nuclei and/or removing the overly segmented nuclei are important for the final segmentation.

The methods described above have been developed to segment cell nuclei or isolated single cells and in both cases the objects of interest appear as mostly well separated solid objects. Our task is much more challenging as we need to isolate each cell instance and accurately define the cell boundaries (as defined by the cell membrane) in whole tissue. Each cell is made up of a solid nucleus surrounded by varying amounts of cytoplasm and bounded by the cell membrane. Cells are generally packed in closely together, so it is often not possible to separate the membrane of one cell from that of its neighbours. This is illustrated in the first row in Figs. 6 and 7. The cell membrane usually appears as a very fine line in the MxIF images and the unavoidable staining variation, which can come from tissue processing, staining and imaging, results in some cells being rendered in the slide with incomplete or broken boundaries (see cells in Region 2 in Fig. 6). This makes the cell boundary hard to define and results in under-segmentation (i.e. failure to split cells properly). Also, weak, non-specific staining in the background or cell interior, may result in over-segmentation (i.e. incorrectly splitting a single cell into more than one object) (see segmentation results by Micro-Net for regions 1 and 2 in Fig. 7). In addition, the visual appearance of each cell varies largely as nuclei are different and they can appear at any location within the cell (see the different cell appearances in Figs. 6 and 7).

A few studies^12,19^ have developed methods for single cell segmentation for MxIF images. Wang et al.^12^ described a method that uses single stained membrane channel images for the fluorescent, phase contrast and differential interference contrast images. First, they used an object-detection based network (i.e. Faster R-CNN^20^) to identify the cell region with a bounding box, and then applied a watershed algorithm^9^ to segment cell boundaries. They demonstrated substantial improvement in the cell count accuracy (i.e. accuracy for identifying individual cells) but using U-net^14^ yielded better pixel-level segmentation accuracy in their study. The most relevant work by Shan et al.^19^ proposed Micro-Net for various segmentation tasks including single cell segmentation for MxIF images using nucleus and membrane channel images as inputs. They used an enhanced network to improve robustness to image noise/variability and reported state-of-the-art performance compared to other widely used deep learning methods. Their method, however, used semantic segmentation and post-processing was required to achieve instance segmentation.

Recently, the field of object-detection based instance segmentation has been substantially advanced. Mask R-CNN^21^, one of the most accurate methods for instance segmentation, was built on the Faster R-CNN. It provides an end-to-end workflow for instance segmentation without the need for post-processing steps by efficiently performing semantic segmentation using FCN in parallel with the object-detection stage. This generic algorithm is widely used in various tasks including segmentation in cellular images^22^. Loh et al.^23^ and Dhieb et al.^24^ demonstrated accurate results using Mask R-CNN for instance segmentation for counting blood cells in microscopic images. Kromp et al.^25^ evaluated U-Net, instance-aware based architectures and two conventional pipelines for nuclear segmentation on complex immunofluorescence images. They found that the instance-aware method (including Mask R-CNN) performed better in the object-based metric (i.e. F1 score) while U-Net based method performed better on the pixel-level based metric (i.e. mean Dice score). Fujita et al.^26^ proposed a method to improve the performance of Mask R-CNN for nuclear segmentation on the 2018 Data Science Bowl dataset. Their method balances the contribution of multiple task losses in training to improve the performance for detecting hard samples. These studies^21,23,24,26,27^ provided valuable insights on implementing Mask-RCNN for microscopy images and demonstrated its effectiveness, particularly for instance identification/detection and segmentation. However, these methods were applied to the tasks of blood cell segmentation and nuclear segmentation which, as mentioned previously, are very different to our task of cell segmentation in tissue MxIF images.

In addition, the literature pointed to the challenges for implementing Mask R-CNN: (1) needing large number of annotated datasets for effective training, and (2) limited generalization capacity for image samples from different datasets or created under different conditions. Manually annotating each cell instance for training is very time consuming, especially for cellular images, which usually have hundreds/thousands of cells in one region of interest (ROI). Even if one could collect a very large number of image samples containing large variabilities and afford to hire a large group to annotate images, it is impossible to include images that represent all possible conditions, nor define annotation standards that fit all use purposes.

Domain adaptation/transfer learning is a method that adapts a model trained for one task for the target task by fine-tuning. For a CNN, this allows it to be pre-trained to learn many feature filters (e.g. edge, intensity, etc.) before fine-tuning. This method has been effective in improving performance in many tasks, especially when the training sample size is not large^28^.

In this article, we present a deep learning-based pipeline for instance cell segmentation for MxIF images of DAPI (a nuclear stain) and Na^+^K^+^ATPase (a membrane stain, referred to here as MEM) stained images by using two-stage domain adaptations to train a Mask R-CNN model. We performed first-stage domain adaptation by fine-tuning the model using a weakly labeled dataset. The second-stage domain adaptation was performed by using a dataset with manually annotated fine labels. We validated our pipeline on two different in-house datasets and one public dataset. The primary contributions of this paper are: (1) description of a two-stage domain adaption method for whole cell segmentation on MxIF images, which allows the model to achieve a human-level performance and a better than state-of-the-art performance validating on different datasets; (2) demonstration that the use of a weakly labeled dataset (using samples from a similar but different domain) for first-stage domain adaption substantially improves the final model performance overall and allows the model to achieve satisfactory results on different domain datasets using a few manually annotated samples for second-stage domain adaptation; (3) presentation of a recursive pipeline for generating high-quality weak labels of cell membrane boundary, which uses a minimum amount of human labor; (4) deployment of our pipeline in Cellprofiler as a plug-in for easy access for both developers and end users.

## Methods

**Figure 1:**
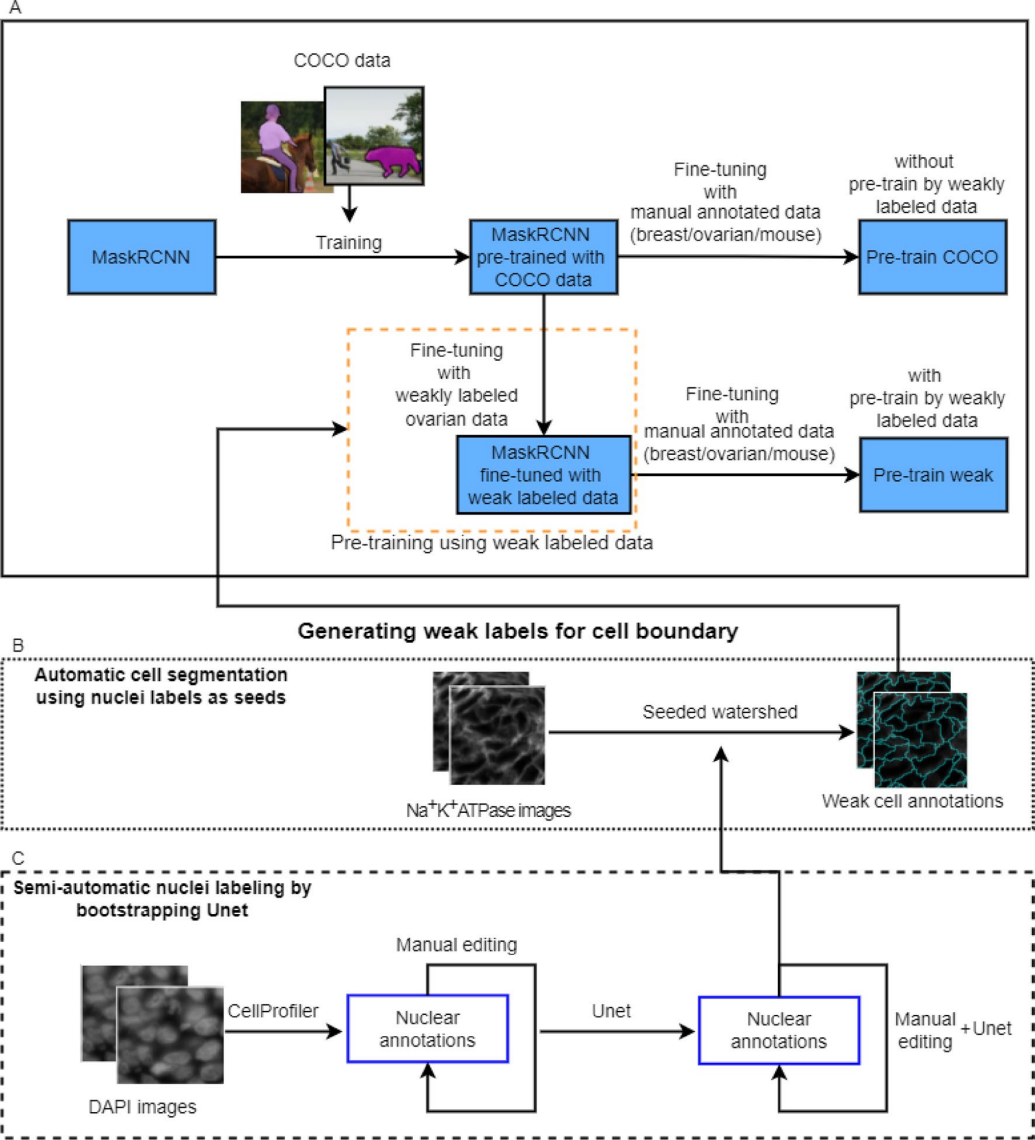
Workflow diagram for methodology: (**A**) (within black solid box) demonstrates the methods of training a Mask R-CNN model using domain adaptation with and without pre-training using weakly labeled ovarian data. The corresponding final models are referred to as *pre-train weak* and *pre-train COCO* respectively. The dotted and dashed boxes (i.e. **B** and **C**) demonstrate the process of generating weak annotations for the cell boundaries: (**B**) describes using the Na^+^K^+^ATPase image to generate weak (i.e. rough estimation) annotations for cell boundaries using seeded watershed by using nuclear labels as seeds. (**C**) demonstrates the semi-automatic nuclear segmentation using bootstrapped U-net after CellProfiler labeling and manual editing of the nuclear annotations. This figure is created using diagrams.net (https://www.diagrams.net/).

Figure 1 is a schematic outline of the workflow for our method. A Mask R-CNN model, pre-trained using natural image data, was trained using two-stage domain adaptation by first fine-tuning using a weakly-labeled dataset (see the workflow for generating weak labels in B and C) followed by fine-tuning with a manual annotated dataset (see the branch resulting *pre-train weak*). For comparison purposes the model was also fine-tuned using the manual labeled data only (see the branch of *pre-train COCO*). The trained system was deployed as a plug-in to the CellProfiler platform. The plug-in deployment packages all the dependencies in an executable for easy access and use (refer to “System development” and “Results” for deployment details).

### Data

This study was approved by our institutional Health Sciences Research Ethics Board, and all methods were performed in accordance with the relevant guidelines and regulations. For the human tissues used in this study, informed consent was obtained from all participants and/or their legal guardian(s). For system validation, a total number of 223 ROIs were used (see the detailed breakdown in Table 1). Three different datasets were used: (1) ovarian cancer, (2) breast cancer, and (3) mouse pancreatic tissue samples (a public dataset used for algorithm comparison purposes). Samples were split 50:50 for training and testing such that samples from the same case/mouse were not used for both training and testing (see the training and testing datasets separately listed in Table 1). For generating the weak labels for the first-stage domain adaptation, a separate dataset (i.e. not overlapped with any of the total of 223 ROIs listed in Table 1) comprising a total number of 210 ROIs (referred as *weak-label-set*) was created by sampling from one ovarian cancer tissue section of a training case. When sampling, we excluded tissue regions that were sampled for creating *O-train*. The *weak-label-set* of 210 ROIs was separated into three groups for the weak label generation (see details in weakly labeled data in the following section); each group contains 26 (*weak set-1*), 16 (*weak set-2*), and 168 ROIs (*weak set-3*) respectively.

**Table 1:** Dataset information (the notation of each dataset is in the brackets).

Dataset	Splitting	Number of cases/mice	Number of cells	Number of ROIs (512 × 512 pixel)
Ovarian cancer	Training (*O-train*)	3	2026	12
	Testing (*O-test1*)	3	2277	12
	Testing (*O-test2*) (multi-observer)	1 (from testing case)	1006	6
	Total	6	5309	30
Breast cancer	Training (*B-train*)	10	2172	20
	Testing (*B-test*)	10	2416	20
	Total	20	4588	40
Mouse pancreatic (external data)	Training (*M-train*)	6	8479	91
	Testing (*M-test*)	4	5583	62
	Total	10	14,062	153
Complete dataset	Training dataset	19	12,677	123
	Testing dataset	17	11,282	100
	Total	36	23,959	223

We performed multiplexing for both the ovarian and breast cancer samples at our laboratory. Ovarian cancer samples were supplied by Dr. Pamela Ohashi’s laboratory (University Health Network, Toronto, ON). A breast cancer tissue microarray (TMA) was purchased from Pantomics (CA, USA). The mouse pancreatic data and expert annotations were downloaded from a publicly available dataset: https://warwick.ac.uk/fac/sci/dcs/research/tia/data/micronet.

**Figure 2:**
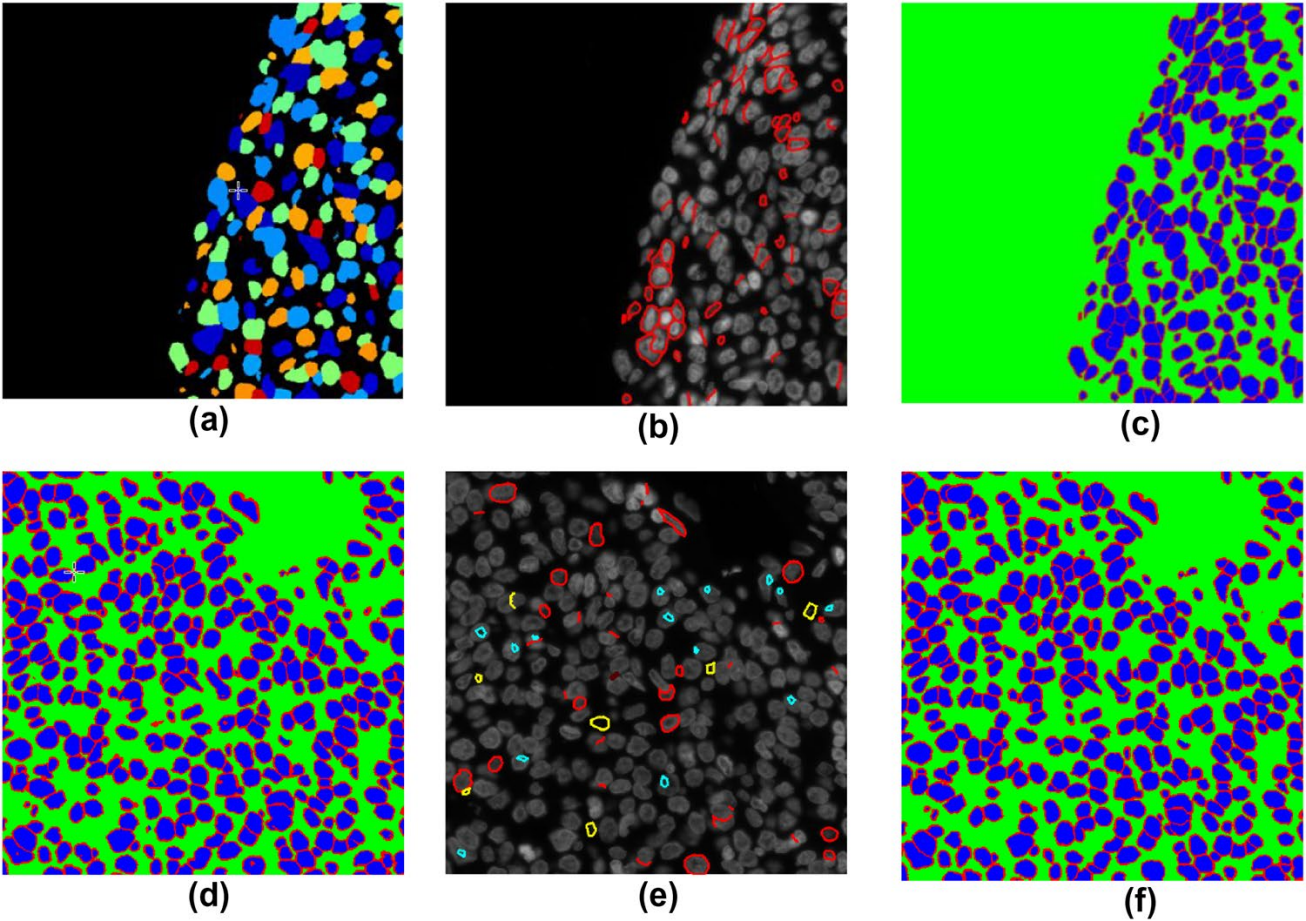
ROI samples (from *weak-label-set*) for nucleus label generation using semi-automatic method. First row images (**a–c**) demonstrate nucleus label generation for the method using Cellprofiler + manual edits. (**a**) Label map generated using Cellprofiler. Different nuclei are labeled by different colors. (**b**) Manual edits that were performed on the DAPI channel image to correct the results in (**a**). Red annotations were added to segment the nuclei. (**c**) Label map generated from (**a**) and (**b**). Second row images (**d**–**f**) demonstrate generating nuclear labels using U-Net + manual edits. (**d**) Label map output from U-Net. (**e**) Manual edits were performed on the DAPI channel image to correct the results in (**d**). (**f**) Label map generated from (**d**) and (**e**). Color codes: In (**c**), (**d**), and (**f**) red—nuclear boundary, blue—interior of nucleus, and green—the background. In (**b**) and (**e**): red represents added lines as nuclear boundaries; yellow contours the nuclear boundaries for removal; cyan contours the nuclei for removal.

**Figure 3:**
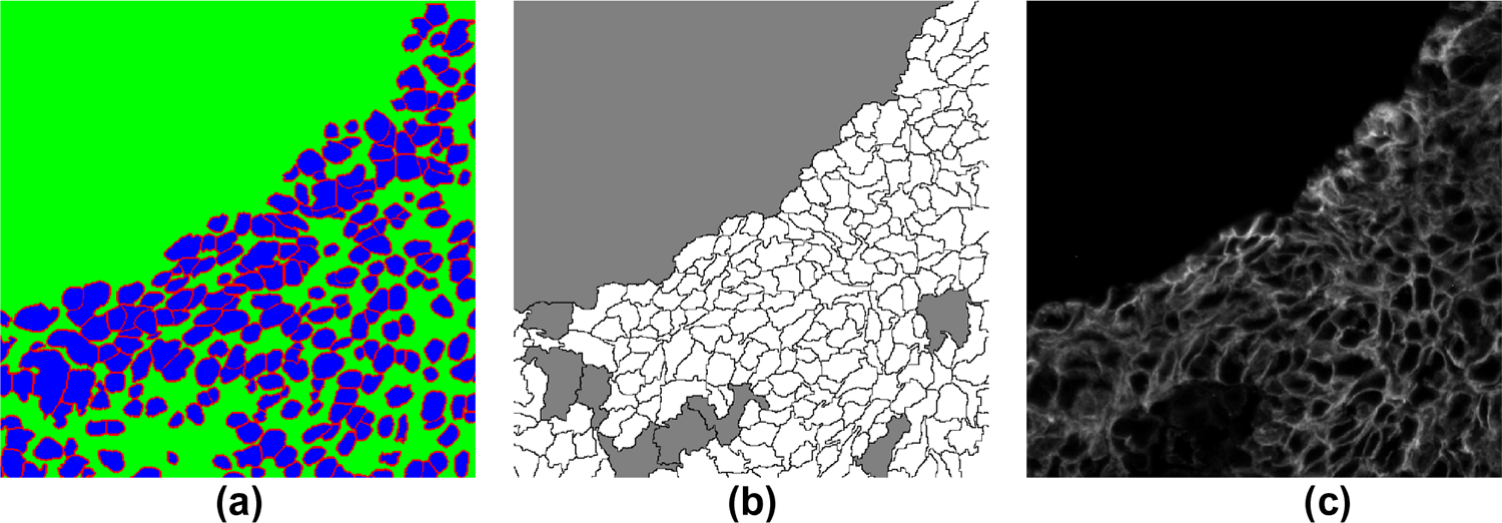
ROI sample (from *weak-label-set*) for cell label generation. (**a**) Nucleus label map in which each nucleus was used as a marker for performing marker-controlled watershed + morphological operations to generate a cell label map (**b**). Color codes in (**b**): white—inner cell regions, black—cell boundaries, and grey—background. (**c**) MEM channel image.

1. Tissue imaging for in-house data
  In this study, we used six ovarian cancer tissue sections from 6 patients and 40 breast cancer cores from a TMA which was created with samples from 20 patients. The tissues were imaged using a prototype Immunofluorescence protein multiplexer (GE Research, Niskayuna NY, USA). The antibodies that were used for sequential staining in this work included: CD3, CD4, CD8, Ki67, CD68, PD1, PDL1. Na^+^K^+^ATPase (MEM) and Ribosomal S6 were used as membranous and cytoplasmic markers respectively and 4′,6-diamidino-2-phenylindole (DAPI) was used as a nuclear marker. The formalin-fixed paraffin-embedded tissues were imaged on glass slides at 0.293 µm/pixel using MxIF and registered using the software, Layers (GE Research, Niskayuna NY, USA).
2. Manual annotated data
  All ROIs (in Table 1) were manually contoured by human observers. The in-house datasets including ovarian and breast cancer tissue samples were annotated by a researcher (Han) trained by a biologist (Cheung) and a pathologist (Liu). *O-test2* (a subset of six ROIs from one section of the testing dataset) was annotated by three observers (i.e. Han, Cheung, and Martel) independently. The annotation was performed to contour the cell boundary, defined by the cell membrane edge seen on the MEM channel (see the white annotation in A–B as an example in Fig. 6). For each ROI, annotation was performed on the Sedeen viewer (www.pathcore.com/sedeen). After conversion of the single channel DAPI channel and MEM channel images from 16-bit to 8-bit, the images were stacked in the R-G-B color space in the order DAPI-MEM-DAPI. The annotator could switch between the DAPI channel, MEM channel, and the stacked color images (SCI) during the process. A polygon tool was used to contour each individual cell. Cells were excluded if they met any of the following conditions: (1) overlapping nuclei; (2) membrane crossing over the nuclei; (3) out of focus nuclei; (4) faintly stained nuclei. The manual annotation process took approximately 2.5 hours per ROI for each observer.
3. External mouse pancreatic data
  The publicly available dataset of mouse pancreatic tissue sections was acquired from https://warwick.ac.uk/fac/sci/dcs/research/tia/data/micronet. The images were generated using a different staining (i.e. E-cadherin) for the membrane. Those tissues were manually contoured by expert biologists^19^. The annotations were carried out using different criteria to those we adopted; each cell was required to be separated from its neighbor by a gap and all cells, including those that were faintly stained or out of focus, were included.
4. Weakly labeled data
  1. Method overview
    Our method for generating weak labels of whole cell boundaries includes (1) nuclear segmentation on DAPI channel images, (2) cell membrane segmentation on the MEM channel images. The workflow can be found in B and C in Fig. 1. In the nuclear segmentation stage (see the process in C in Fig. 1), we created a pipeline using conventional algorithms in CellProfiler to segment nuclei on a small set of samples (i.e. *weak set-1*). To improve the label quality to train a U-Net, a human observer participated in editing/correcting the segmentation results (e.g. splitting touching/overlapped objects (Fig. 2b)). We then trained a U-Net using the *weak set-1*. The trained model was used to generate nuclear labels for the *weak set-2*. A second round of human editing helped in correcting U-Net generated labels (Fig. 2d–f). Finally, we trained the U-Net using both *weak set-1* and *weak set-2* and used the trained U-Net to label nuclei for the *weak set-3*. Given the nuclear labels from the *weak-label-set*, we then produced cell boundary weak labels using marker-controlled watershed (B in Fig. 1). In such a recursive manner for human-involved editing and U-Net training, on successive iterations the human observer only needs to correct a small number of nuclei that were incorrectly segmented/labeled. The details of the methods are described in the following sections.
  2. Semi-automatic nucleus segmentation
    For the *weak set-1*, nuclei were segmented on the DAPI channel images using Otsu’s method^29^ for thresholding followed by seeded watershed performed in CellProfiler (see result example in (a) in Fig. 2). Segmentation results were then reviewed and manually edited using Sedeen viewer^30^ (Fig. 2b). The DAPI channel images of the *weak set-1* and the nuclear segmentation results (Fig. 2c) were used to train a U-Net^14^ for nuclear segmentation. Segmentation was then performed on the *weak set-2* of the DAPI channel images using the trained U-Net. These segmentation results were reviewed and edited manually (Fig. 2d–f). Both *weak set-1* and *weak set-2* were used to train another U-Net. Nuclear segmentation was then conducted on *weak set-3* using that trained model.
    Our U-Net^14^ implementation is based on the implementation (https://github.com/carpenterlab/unet4nuclei/) described by Caicedo et al.^31^ for nuclear segmentation. We adapted the code to implement it using Pytorch. We used DAPI channel images as input, and three-class label maps (i.e. color images in which red labels the nuclear boundary pixels, blue labels pixels within the nucleus, and the background pixels are shown in green) as ground truth for training (see Fig. 2c,f).
  3. Automatic cell boundary segmentation using marker-controlled watershed
    For the *weak-label-set*, nuclear segmentation was performed as described above. Next, cell segmentation (see results in Fig. 3b) was performed on the MEM channel (Fig. 3c) images using seeded watershed^9^ (see B in Fig. 1). The nuclear segmentation results (see Fig. 3a) include three labels: (1) inner nucleus; (2) nuclear boundary; (3) background. A morphological erosion was performed on the inner nucleus using a 3 × 3 disk to reduce the size of the marker. The resulting regions were used as seeds to perform watershed segmentation of the cells. Some segmented regions were found to correspond to large background regions, therefore, a size filter was used to remove any regions ≥ 1400 pixels (see grey regions in Fig. 3b).

### System development

Our proposed method (*pre-train weak*) used the following steps (see workflow in Fig. 1). Initially, the Mask R-CNN model was pre-trained for instance segmentation using a large dataset of natural images (i.e. the MS COCO dataset^32^). A two-stage domain adaptation was then performed: (1) fine-tuning with *weak-label-set*; (2) fine-tuning with manual annotated datasets. Weak cell boundary labels were generated using a semi-automatic method as described above (see steps in B and C in Fig. 1). For comparison purposes, experiments were also performed on a model without pre-training using the weakly labeled dataset (*pre-train COCO*) (see Fig. 1). In the experiment using a public dataset (see details in experiment design below), a model (*pre-train breast*) was fine-tuned from the final model trained for the breast cancer dataset.

**Figure 4:**
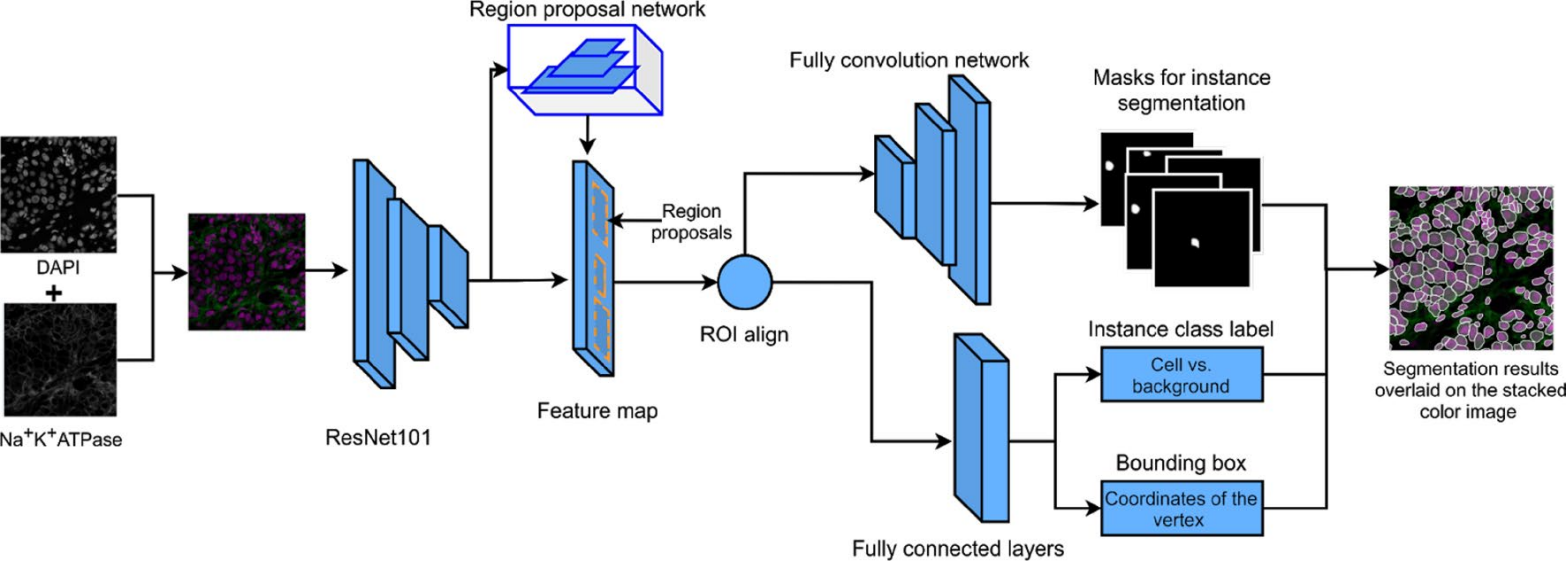
Mask R-CNN workflow for single cell segmentation of multiplexed images. The input images are the color stacked images from the DAPI and Na^+^K^+^ATPase -stained single channel images. The input went through the backbone (i.e. ResNet101) to form the feature map. Another branch went through the region proposal network to generate the instance candidate (i.e. region proposals) on the feature map. The following branches are: (1) full convolutional network for segmentation for the proposed instances, (2) fully connected layers for classification and bounding box regression for the instances. This figure is created using diagrams.net (https://www.diagrams.net/).

Mask R-CNN^21^ is a deep learning architecture for instance segmentation (see workflow in Fig. 4). There are two stages: (1) generating the proposals for the regions where the objects might exist using a region proposal network (RPN) described by Lin et al.^33^; (2) based on the proposals, performing classification (i.e. classifying each proposal as object labels), refining the bounding boxes of the proposal, and segmenting the object. The network structure uses a feature pyramid network^33^ (FPN) backbone, which includes a bottom-up pathway and up-bottom pathway. The bottom-up pathway can be a convolutional neural network (CNN) such as ResNet-50, or ResNet-101^34^.

The Mask R-CNN implementation^27^ is based on the implementation by Matterport Inc. released under an MIT License. SCIs were used as input images. Stacked binary masks (i.e. cells vs. backgrounds) served as ground truth (see the example of binary mask in Fig. 4), and these were extracted from the cell labels as described in the previous section.

During training, the model was initialized with the pre-trained weights generated using the MSCOCO dataset^32^ (https://github.com/matterport/Mask_RCNN/releases/download/v1.0/mask_rcnn_coco.h5). ResNet-101 was used as the backbone structure. To perform cell segmentation, the network heads were first trained for 20 epochs, and then all layers were trained for 40 epochs. Binary cross entropy was used as the multi-loss function, and stochastic gradient descent (SGD) as the optimizer. The training parameters are: batch size = 6, learning rate = 0.0001, weight decay = 0.0001, momentum = 0.9. The gradients were clipped to 5.0 to prevent gradient explosion. The input images were scaled up by a factor of two using nearest neighbor interpolation and normalized across channels. The input images were augmented randomly using the following methods: (1) flipping vertically/horizontally for 50% of all images, (2) blurring images using a Gaussian kernel with sigma of 5.0, (3) multiplying by a random value between 0.8 to 1.5 to darken/brighten input images, (4) randomly rotating the images by an angle of 90°, 180° or 270° using affine transform. The model was fine-tuned using the identical setup as the training stage.

The method was deployed as a CellProfiler plug-in for cell segmentation for MxIF images. As analysis pipelines also require nucleus segmentation, a separate nuclear segmentation model was trained with the 2018 Data Science Bowl nucleus dataset^35^ and fine-tuned with the dataset with nuclear segmentation labels that were used for generating weak cell boundary labels. The same setup was used for training and fine-tuning as described above.

Our implementation for seeded watershed used Matlab 2018b (The Mathworks, Natick, MA). The seeded watershed algorithm and manual annotation were performed on a PC with AMD Ryzen 5 2600 CPU, 16 GB memory, and Gigabyte GeForce RTX 2070 8 GB GPU. Our implementation for U-Net, training and fine-tuning of Mask R-CNN were performed on our server using a single Nvidia Titan XP GPU with a setup of dual Intel Xeon E5-2660 CPU and 128 GB memory. Experimental validations were performed using Compute Canada (www.computecanada.ca) with one node using 48 CPU cores.

Our system was deployed using the Flask framework^36^, which included the plug-in and the executable components. The plug-in is a Python script written in CellProfiler plug-in specified format. The executable was compiled using Pyinstaller-4.1 with our source code and run-time environment dependencies.

### Experimental design

#### Experiment validating against multi-observer annotations

The system was trained using *O-train* and validated on the *O-test2* against each observer’s annotation respectively and the results were averaged. To evaluate the annotation agreement between different observers as a baseline for comparison, we validated the annotations between all pairs of observers and averaged the pairwise results.

In order to evaluate the potential impact from the observer annotation, we also conducted a comparative analysis on *O-test2*. We compare the system results when validating against the researcher’s annotation and the expert’s annotation. We also compare those results to the inter-observer agreement between the researcher (Han) and expert (Cheung).

#### Experiments validating on three different datasets using different training sample sizes

To evaluate the generalization capacity of the system, our methods were validated against manual annotations using (1) the *O-test1*, (2) *B-test*, and (3) the *M-test*. For each dataset, the experiment was performed using different training sample sizes from the training datasets (i.e. *O-train*, *B-train*, *M-train*) and validated on the corresponding testing datasets (Table 1). Specifically, for example, for ovarian cancer tissue datasets, the model was trained using samples from *O-train* and validated on *O-test1*. Similarly, for breast cancer dataset, the model was trained using samples from *B-train* and validated on *B-test*. Our methods of *pre-train weak* and *pre-train COCO* were compared on our in-house datasets. The methods (i.e. *pre-train weak*, *pre-train COCO* and *pre-train breast*) were compared to Micro-Net (state-of-the-art), and U-net using the public dataset. The models were trained with samples from *M-train* and validated on *M-test*. The direct comparison to Micro-Net and U-net can be found when using a training sample size of 72 ROIs, which is the sample size Shan et al.^19^ used in their study.

#### Error metrics

Object-Dice (OD) and object-Hausdorff (OH) were computed as metrics to measure the system performance^37^. The Dice coefficient^38^ measures how well the segmented region matches the ground truth. Hausdorff distance^39^ measures the longest distance between the segmentation boundary and the ground truth boundary, which gives the largest misaligned distance. Computing those metrics at the object-level takes object identification into consideration. For example, touching cells that are well segmented as a whole but not properly separated can have high value of Dice but lower value of Object-Dice weighted by the cell size.

### Approval for human experiments

This study was approved by our institutional Health Sciences Research Ethics Board (Sunnybrook Research Institute Health Sciences Research Ethics Board, Toronto, Ontario, Canada).

## Results

### Experiment validating against multi-observer annotations

The results are shown in Table 2. *Pre-train weak* yielded comparable results to the multi-observer agreement in both metrics of OD and OH, while *pre-train COCO* showed inferior results with slightly lower OD and higher OH.

**Table 2:** Multi-observer validation results for *O-test2* dataset.

Methods	Object dice (OD)	Object hausdorff (OH) (pixel)
Multi-observer	0.76 ± 0.03	10.03 ± 1.26
Pre-train weak	0.76 ± 0.04	9.25 ± 0.86
Pre-train COCO	0.73 ± 0.03	10.66 ± 1.03

**Figure 5:**
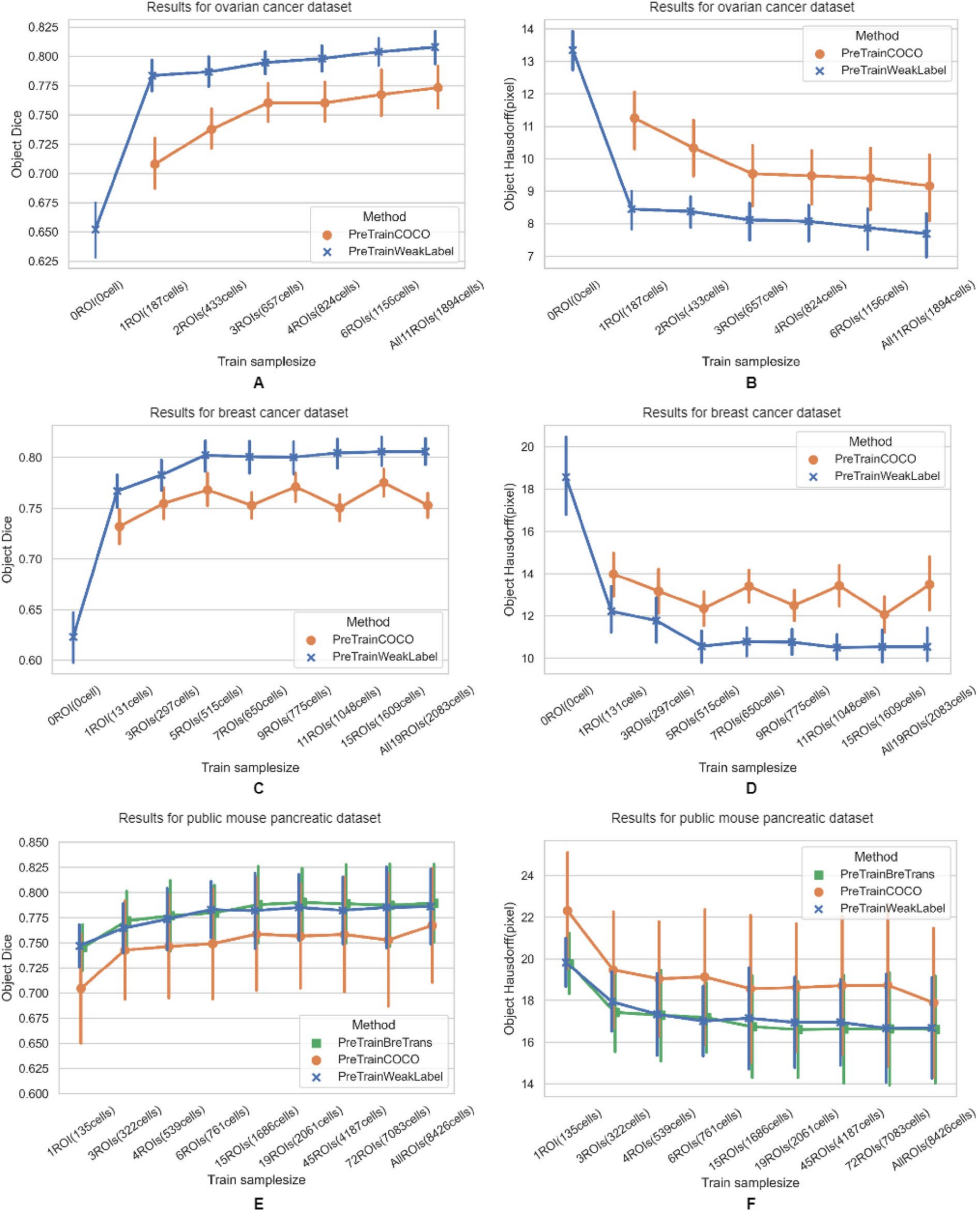
Experiment results on three different datasets using different training sample sizes for training. Left and right columns are OD vs. training sample size and OH vs. training sample size respectively. (**A**, **B**), (**C**, **D**), and (**E**, **F**) are the results using the ovarian cancer dataset, breast cancer dataset, and public mouse pancreatic dataset respectively. Color code in the plots represents different methods.

Comparative results are shown in Table 3. The annotation agreement between the researcher (Han) and expert (Cheung) are close to the multi-observer agreement (Table 2). For *pre-train weak*, the results validating against Cheung are close to the multi-observer agreement (Table 2). When validating against Han’s annotation, which were used for second-stage domain adaptation, the results show higher agreement (OD of 0.80 and OH of 7.86). This result is similar to the results on other datasets with single observer annotations (see results reported in Fig. 5). In Table 3, the differences between two pairwise results are within the range of the standard deviations of the two. Based on our observation including two expert observers (Liu and Cheung), the primary disagreements between the human observers and model vs. each observer come from the borderline cases where controversy may arise due to the excluding rules.

**Table 3:** Comparative results for *O-test2* dataset for researcher (Han) and expert (Cheung) annotations.

Pairwise validation	Object dice (OD)	Object hausdorff (OH) (pixel)
Han vs. Cheung	0.77 ± 0.03	9.14 ± 1.11
Pre-train weak vs. Han	0.80 ± 0.03	7.86 ± 0.76
Pre-train weak vs. Cheung	0.75 ± 0.04	9.92 ± 1.35

### Experiments validating on three different datasets using different training sample sizes

The results are shown in Fig. 5. In general, *pre-train weak* (blue plots) yields higher OD and lower OH than the *pre-train COCO* (orange plots) in all the experiments, suggesting that it provides superior performance. The performance differences between the two methods are larger when the training sample sizes are smaller for the experiments using ovarian cancer and external datasets. Compared to *pre-train weak*, for both metrics, *pre-train COCO* has higher standard deviations and shows larger variation (i.e. changes between the points) when using different training sample sizes.

For all methods, we observed increased OD and decreased OH as training sample size increases, and the two-stage domain adaptation methods (i.e. *pre-train weak* and *pre-train breast*) are less sensitive to training sample size with a boosted performance than *pre-train COCO*. The *pre-train weak* method achieves close to optimal OD and OH when using training sample sizes of more than 600 cells for all experiments. This also applies to the *pre-train breast* method applied to the public dataset. In contrast, the *pre-train COCO* method requires a larger training sample size and there are large variations in OD and OH. In the experiments using ovarian and breast cancer datasets, the *pre-train weak* using a training sample of one ROI achieves higher OD and lower OH than *pre-train COCO* using all the available training data (A–D in Fig. 5). Similarly, *pre-train weak* used three ROIs for training to achieve better performance than *pre-train COCO* using all the training samples in the experiment using the public dataset (E and F in Fig. 5). We note that the available training sample size in the public dataset includes 90 ROIs, which contains 8426 annotated cells.

For the experiment using publicly available data, *pre-train breast* and *pre-train weak* have similar ODs and OHs for all training sample sizes (E–F in Fig. 5). When using a training sample size of 72 ROIs for direct comparison, both methods have superior performance compared to Micro-Net (Table 4). The performance superiority remains when using a sample size as small as one ROI for training (Fig. 5 and Table 4). *Pre-train COCO* also has a slightly higher OD and a much lower OH than Micro-Net using the training sample size of 72. All methods using Mask R-CNN, regardless of training sample sizes, have lower OHs than Micro-Net using 72 ROIs for training (Fig. 5 and Table 4). In addition, all methods, regardless of training sample sizes, show substantially higher OD and lower OH than U-net as reported by Shan et al.^19^ (OD of 0.67; OH of 40.39) using training sample size of 72ROIs (Fig. 5 and Table 4).

**Table 4:** Validation results for *M-test* dataset using training sample size of 72 ROIs.

Methods	Object dice (OD)	Object hausdorff (OH) (pixel)
Micro-Net	0.74 ± 0.07	27.53 ± 11.36
Pre-train weak	0.79 ± 0.05	16.63 ± 3.01
Pre-train COCO	0.75 ± 0.08	18.73 ± 4.51
Pre-train breast	0.79 ± 0.05	16.67 ± 3.00

### Example visual results from the experiments

**Figure 6:**
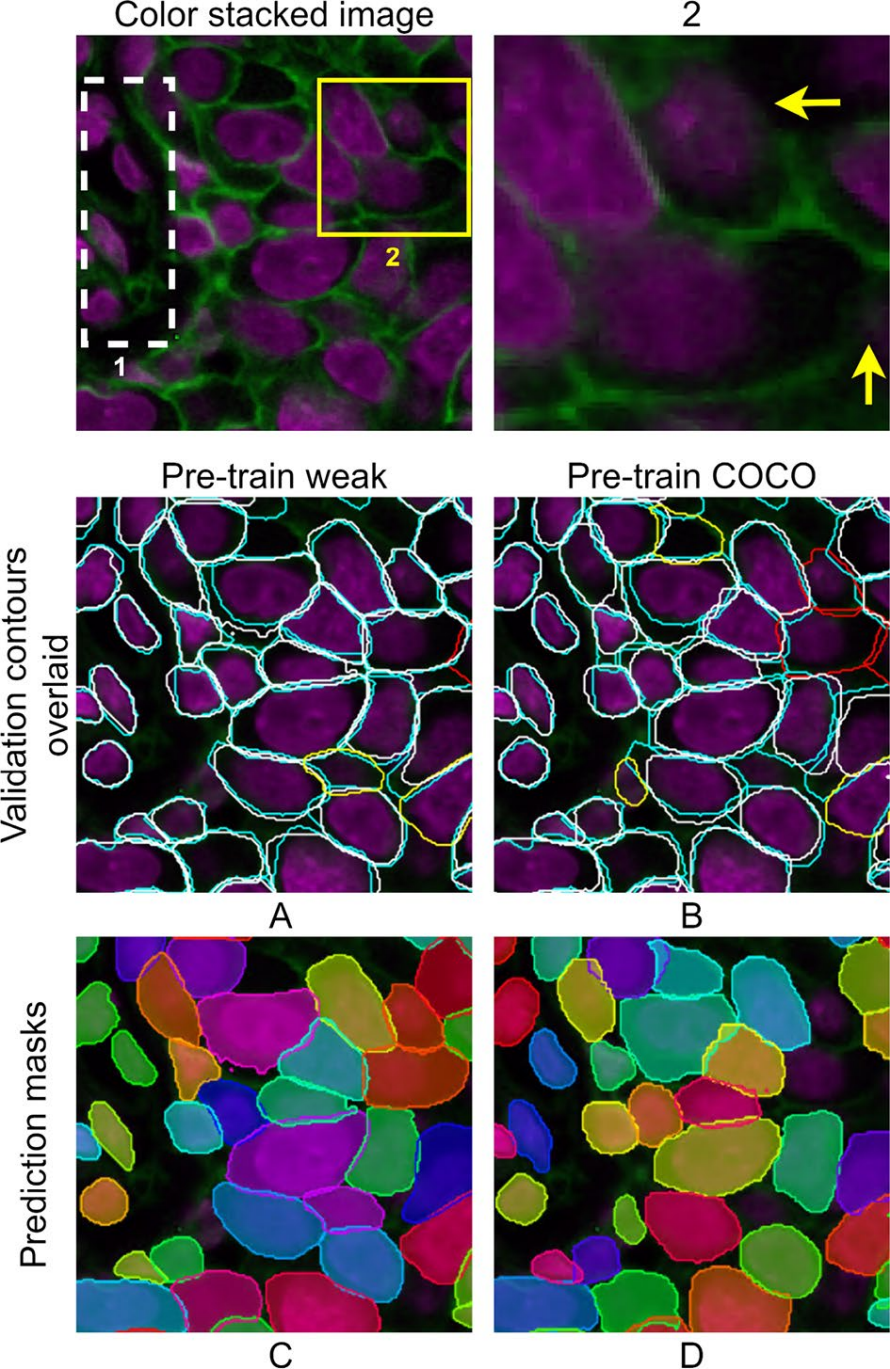
Visual results for the example image of a breast cancer tissue sample. First row is a color stacked image from DAPI and Na^+^K^+^ATPase-stained images. The Na^+^K^+^ATPase channel is enhanced for visualization. Yellow square highlighted Region 2 is zoomed in at right. White dashed box 1 contains cells that have tight boundaries. (**A**,**B**) show the system output contours and manual contours overlaid on the color image. White: system prediction. Cyan: manual annotation. Red: False negative. Yellow: False positive. (**C**,**D**) are prediction maps by the systems. Each contour mask is shown with a different color. This figure is created using diagrams.net (https://www.diagrams.net/).

Figure 6 shows an example of our experimental results. We observe that most system outputs are closely aligned to the manual annotation, with *pre-train weak* showing slightly better alignment than the *pre-train COCO* (see cyan and white contours in A–B). Each cell is contoured by the system independently without broken contours (see color masks in C-D). Cells that have very tight boundaries (cells in the white dashed box 1) are also contoured by both *pre-train weak* and *pre-train COCO* (see cells in the white dashed box 1 in A–B). It was found that *pre-train weak* yields lower numbers of FNs and FPs than *pre-train COCO* (see FNs by red contours and FPs by yellow contours in A–B), especially at the region where the cells are tightly packed with weak boundary stains (see cell boundaries indicated by yellow arrows in Region 2 and result maps A–B).

**Figure 7:**
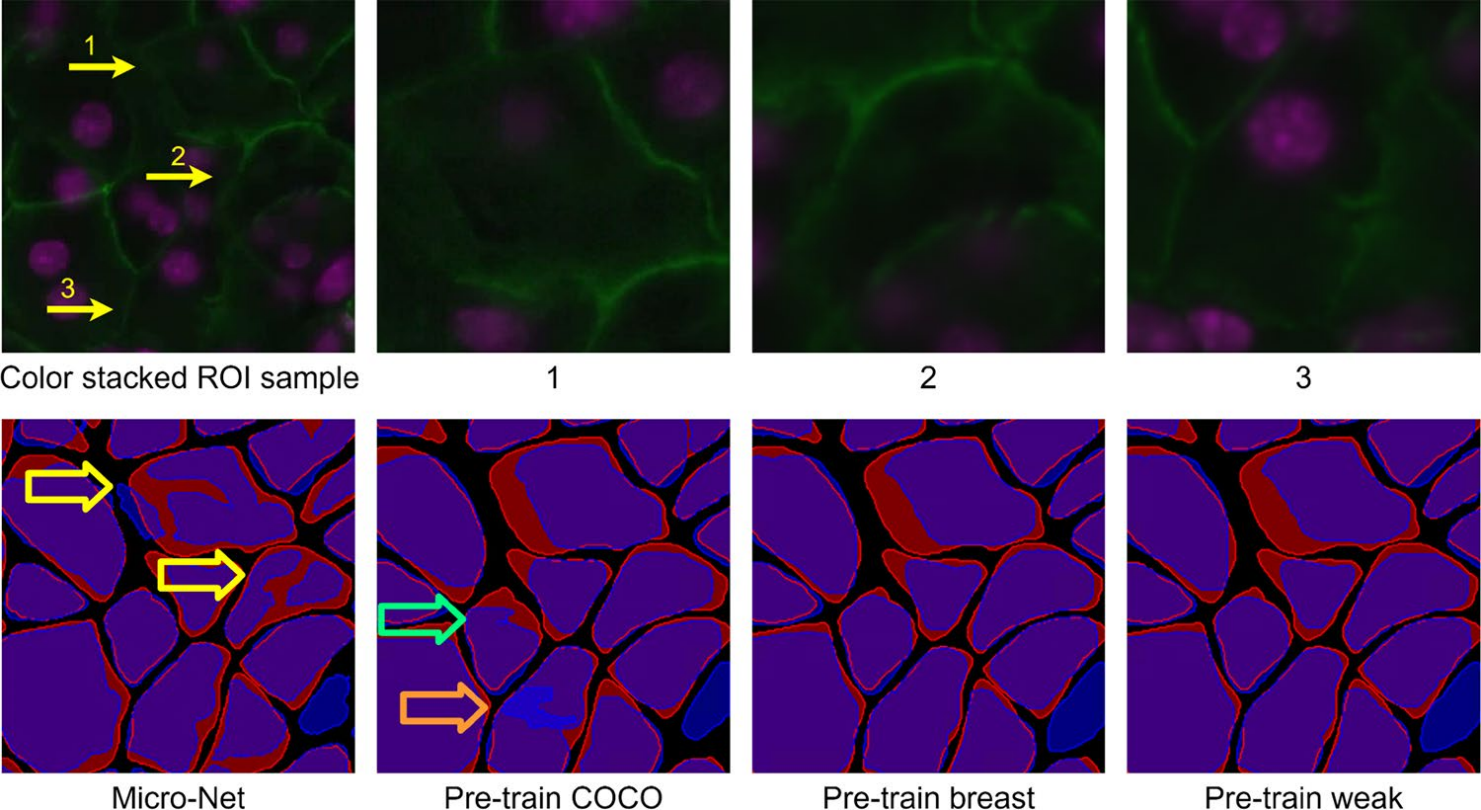
Visual results for an example of a mouse pancreatic tissue sample. The first row shows the color stacked image from DAPI and E-cadherin (Ecad) stained images 1, 2 and 3 are regions highlighted by the yellow arrows. Those regions are in a zoomed-in view at the right. The second row shows the results using different methods. Red: contour mask generated from manual annotation. Blue: contour mask generated from system outputs. Purple: overlapped region of manual annotations and system outputs. This figure is created using diagrams.net (https://www.diagrams.net/).

Figure 7 shows an example of our experiments on the public dataset. In general, all outputs are closely aligned with manual annotations. *Pre-train weak* and *pre-train breast* show similar performance with the closest alignment to the manual annotations. In contrast, Micro-Net has cells that are overly segmented, and those masks show irregular and fragmental shapes (see Region 1 and 2 in the result maps indicated by yellow arrows for comparison). This results in a large value of Hausdorff distance. *Pre-train COCO* shows a lower degree of alignment to manual annotation than *pre-train breast* and *pre-train weak* (see the example indicated by the green arrow in result maps). It also has an overly segmented cell (see Region 3, and cell indicated by the orange arrow in the *pre-train COCO* result map).

### Implementation deployment

**Figure 8:**
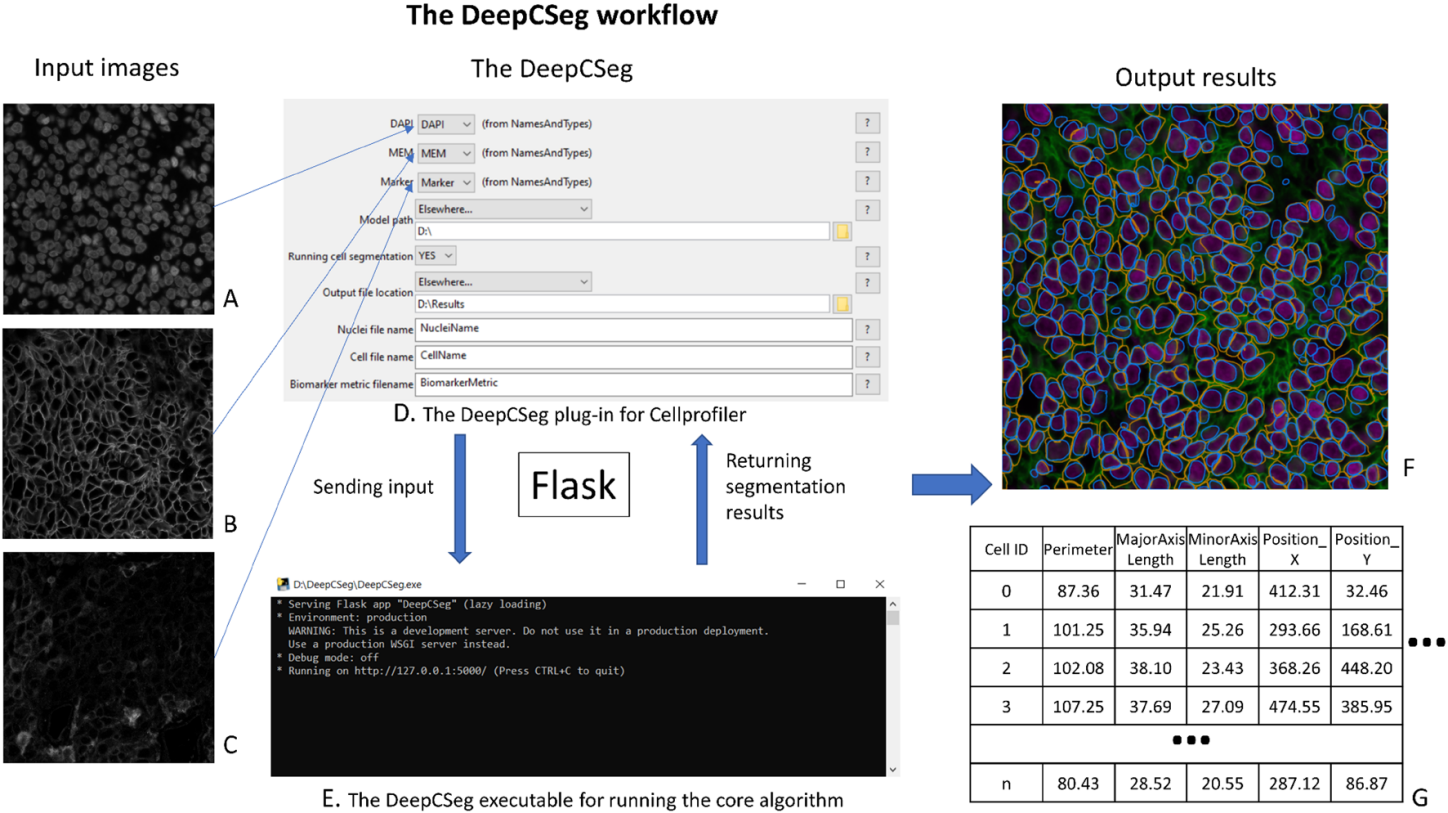
The DeepCSeg plug-in workflow. The input images are the immunofluorescence multiplexed images stained with (**A**). DAPI, (**B**) Na + K + ATPase, and (**C**) pan-cytokeratin. There are two components in DeepCSeg: (**D**) the plug-in for the CellProfiler and (**E**) the executable for implementing the core algorithm. Flask is the framework that is used for the communication between the two components. (**F**) shows the output results for whole cell and nuclear segmentation on the color image, which is synthesized from (**A**) and (**B**) in the R-G-B color mode. The yellow contours identify the cell boundaries, and the blue contours identify the nuclear boundaries. (**G**) The quantitative measurement (e.g. cell central location, size, mean intensities etc.) for each cell for the testing biomarker image (**C**), which is computed using the whole cell and nuclear segmentation results. This figure is created using diagrams.net (https://www.diagrams.net/).

The workflow is shown in Fig. 8. We set up the inputs and outputs in the CellProfiler interface (D). The input images are DAPI (a nuclear stain) and Na^+^K^+^ATPase (a membrane stain) channel images for nuclear and cell segmentation. Pan-cytokeratin channel image (C) is the image for cellular profiling as an example. Once the executable is successfully launched (E), the “Analyze image” button is clicked to run the algorithm. The segmentation results are overlaid in the color image (F). These results were applied to the pan-cytokeratin channel image for cellular profiling and image is presented (G). Users can profile images from multiple channels simultaneously by adding more images as input. A detailed demo is available at: https://youtu.be/gpLDjPQJF8Q. The setup tutorial is available at: https://www.youtube.com/watch?v=sirRJc-A4tc. The package is publicly available at: https://drive.google.com/drive/folders/1WBYFH9bf89s-xjQNZHKSGdFov08h0iFG?usp=sharing. The source code is available at: https://github.com/WenchaoHanSRI/DeepCSeg.

The source code and testing data for the three experiments is available at: https://github.com/WenchaoHanSRI/Cell-Segmentation/tree/main/Mask_RCNN-master.

## Discussion

We have described a two-stage domain adaptation pipeline to train a Mask R-CNN for instance cell segmentation of MxIF images using a weakly labeled dataset for pre-training. The trained model provides end-to-end instance segmentation without the need for any pre- and post-processing steps. The segmentation results were validated against three different manually annotated datasets with a performance that matches multi-observer agreement on the ovarian cancer dataset (Table 2), and a better performance than the state-of-the-art on the public mouse pancreatic dataset (E–F in Fig. 5 and Table 4). We deployed our model to a widely used software platform, CellProfiler, as a plug-in for easy access. The plug-in runs using an executable backend without the need for installing any software dependencies. It performs instance nuclear and cell segmentation followed by cellular profiling for the target image channel(s). The plug-in demonstrates the potential for supporting more efficient immunoprofiling using MxIF images (Fig. 8).

Our method using two-stage domain adaptation boosted the model performance, and the advantage is more obvious when the training sample size is small. This is demonstrated in Fig. 5 where the *pre-train weak* network (blue plots) shows higher OD and lower OH than the *pre-train COCO* network (orange plots) in all experiments; both models used the same network (i.e. Mask R-CNN) but were trained differently. In the experiment using the public dataset, *pre-train COCO* shows a similar OD to the method of Shan et al.^19^ regardless of the training sample size. In contrast, the two-stage domain adaptation methods showed a better performance than the state-of-the-art even when only a few ROIs were used for fine-tuning (E–F in Fig. 5 and Table 4). In addition, the two-stage domain adaptation approach showed more robust and consistent performance than the single domain adaptation method (i.e. *pre-train COCO*). The volatility of the *pre-train COCO* results may be due to the uneven distribution of different training samples, and it is possible that our method makes the model less sensitive to the selection of training samples by using a large number of weakly labeled samples for pre-training.

Our method of generating weak labels is efficient for the first-stage domain adaptation, which creates a large number of weakly labeled data that have sufficient quality for pre-training. First, comparing to the existing methods (including conventional methods and a deep learning-based approach), we used a combination of conventional algorithms and a deep learning model (i.e. U-Net) with a recursive manual label correction process. Our method takes advantage of the low computation cost of conventional methods to create a set of coarse labels for a small number of samples. We also take the advantage of batch processing capacity and accurate segmentation performance using U-Net to generate more labels. The recursive process of both manual correction and U-Net training improved the label quality (see example images from Figs. 2 and 3) and increased training sample size with a minimum labor cost involved. Also, generating these weak labels is much easier than manual annotation. For one ROI that contains approximately 200 cells, it takes about 3 hours to annotate. In contrast, the weak labels take about 3 minutes for manual editing. Importantly, the mouse pancreatic data experiment shows that pre-training using these weak labels performs equally well as manual labels (E and F in Fig. 5*pre-train weak* vs. *pre-train breast* which was pre-trained with 2000 manual annotated cells). We speculate that the first-stage fine-tuning effectively trained the network for identifying cell objects. This can also explain our observation that the primary difference in results between *pre-train weak* and *pre-train COCO* is the number of FNs. *Pre-train COCO* was not pre-trained with more samples, therefore, has more FNs (A-B in Fig. 6). Finally, pre-training the network with weakly labelled data from one dataset, allows the model to be fine-tuned using a very small amount of labelled data (i.e. approximately 600 cells) from the target dataset to achieve a performance that is close to optimal (obtained when fine-tuning used more than 8000 cells) as shown in Fig. 5, even when the annotations are slightly different to those in the initial dataset (i.e. difference between mouse annotations that do not touch^19^ vs. our in-house dataset annotations that are touching). It is extremely impractical to annotate thousands of cells for each task and our method enables easier adaptation by using a very small sample size of manually annotated data for fine-tuning.

Our method and experimental results should be interpreted with the following limitations. First, although our study used a large sample size from three different datasets, the sample size is still limited and further improvements in performance may be achievable when a larger sample size is available for hyper-parameter tuning. Second, although we used three different datasets, which includes annotations done by four different observers, we cannot rule out annotation bias as each dataset was primarily annotated by a single observer. We expect the model may be biased toward the annotator whose annotation is used for second-stage fine-tuning (Table 3 and Fig. 5). Our comparative analysis indicated that the bias may not substantially impact results even when validating against a different annotation. For example, in comparing the OD and OH between the two observers, and model vs. one of the observers in Table 3, the differences between two pairwise results are seen to be within the range of the standard deviations of the two. In addition, based on the observation on the samples from the comparative analysis, the disagreement primarily arises from the “include/exclude” rules. In application, since the users may need to annotate a few ROIs for the second stage domain adaptation for their specific task, our reported results should still validly reflect the model performance in application. We have deployed our method in an open-source platform and the source code is publicly available. We recommend that this tool be validated by a broader group of users in the near future. Third, to compare our method to the most relevant works^12,19^, it is not immediately clear if Mask R-CNN is superior to Micro-Net for the experimental task because Micro-Net was not pre-trained by the MS COCO dataset nor the weakly labeled data. However, the use of Mask R-CNN is suitable for our need for end-to-end instance-level cell segmentation. Finally, in order to make the model more generic, our results did not include any post-processing steps, which usually requires manually setting hyper-parameters. In practice, post-processing steps may be helpful to further improve the performance for specific tasks.

## Data Availability

All of our testing datasets with manual annotation indices are available via: https://github.com/WenchaoHanSRI/Cell-Segmentation/tree/main/Mask_RCNN-master. The training datasets will be publicly available once the paper is accepted for publication.
